# Detecting and Grading Valve-Plate Wear in an Axial Piston Pump from Pressure and Vibration Signals: Within-Campaign Separability Versus Leave-One-Condition-Out Generalisation

**DOI:** 10.3390/s26216703

**Published:** 2026-10-23

**Authors:** Fabian H. Diaz Palencia, Carlos Borrás Pinilla, Pedro J. Diaz Guerrero

**Affiliations:** School of Mechanical Engineering, Universidad Industrial de Santander (UIS), Bucaramanga 680002, Colombia; cborras@uis.edu.co (C.B.P.); pjdiaz@uis.edu.co (P.J.D.G.)

**Keywords:** axial piston pump, valve-plate wear, volumetric efficiency, multi-sensor condition monitoring, fault diagnosis, support vector machine, leave-one-condition-out validation, data leakage, generalisation

## Abstract

Valve-plate wear in axial piston pumps reduces volumetric efficiency, and data-driven classifiers often report near-perfect accuracy in grading it. This paper asks how much of such accuracy reflects the fault rather than the recording. Outlet and precharge pressure, leakage flow and housing vibration at four points were recorded on a test bench for five induced wear states (estimated volumetric efficiency 94.9–69.5%) at an unloaded and a loaded (≈700 psi) operating point, in a single campaign. Statistical features were classified with support vector machines, random forests and shallow neural networks under protocols of increasing difficulty. Record-level cross-validation separates the five states with 94–100% accuracy from pressure or from any single accelerometer. Leave-one-condition-out validation shows that only a coarse decision generalises to a wear state absent from training: significant degradation (efficiency below about 80%, where the pump loses its precharge pressure) is detected by the pressure channels at both operating points and by three accelerometers at the loaded point, whereas the fine grading and a continuous efficiency estimate (mean absolute error 3.6–7.0 points) do not transfer, nor do models between operating points. The results are delimited to one campaign with one mounting per wear state; multi-session validation on naturally worn components is required.

## 1. Introduction

Axial piston pumps transmit power in mobile machinery, stationary systems and process industries, and the performance of the whole installation depends on their condition. Companies therefore invest heavily in predictive, preventive and corrective maintenance to extend service life and reduce failures. The nonlinear behaviour of these pumps, coupled across several physical domains, makes the timely detection and identification of incipient faults difficult [1].

Among the components of an axial piston pump, the valve plate (and the bearing plate it runs against) is one of the parts most exposed to wear. As the plate wears, the sealing lands lose material, internal leakage grows and the pump loses volumetric efficiency; the delivered flow and the pressure the pump can sustain both drop, the oil temperature rises and more torque is required to drive the pump [2,3]. If the loss of efficiency is not caught early, it eventually compromises the actuators fed by the pump. A condition-based maintenance (CBM) programme for such a pump therefore needs a method that not only flags the fault but also grades how far it has progressed—and, ideally, a clear statement of the operating conditions under which that grading is reliable.

Fault detection and diagnosis (FDD) systems follow two broad approaches [4]. Model-based methods build a mathematical model of the machine and compare its predicted and measured behaviour to generate residuals; their effectiveness depends on how well the model reproduces the dynamics of the system [5,6]. Data-based methods, in contrast, learn patterns from signals collected by a monitoring system and flag deviations from the healthy pattern. The data-based approach needs much less analytical knowledge, at the cost of depending on the amount and quality of the operating data. Learning classifiers—neural networks and support vector machines (SVMs)—are the tools most used and recommended for this purpose, in applications ranging from nuclear-plant equipment [7] to photovoltaic installations [8], with SVM standing out for condition monitoring because of its solid mathematical basis, its structural-risk-minimisation principle (which helps when training data are scarce) and the availability of efficient solvers [9,10].

Research on the diagnosis of pump faults and wear can be grouped into four lines, each with its own contribution and its own limitation.

Physically based indicators. The first line extracts indicators with a direct physical meaning from pressure, flow, shaft speed or vibration. Early examples are the pulsating-parameter method [11], wavelet analysis of the outlet pressure and wavelet-packet residuals [12,13,14], the energy distribution of the pump vibration [15] and intermittent-chaos statistics of the vibration signal [16]; the fluctuation of the instantaneous angular speed of the shaft reveals pump faults [17] and has recently been used to follow normal, slight and severe valve-plate wear under non-stationary operating conditions [18]; and model-based approaches either predict the loss of volumetric efficiency from friction and wear tests of the piston pair [19] or generate residuals from a model of the pump [5]. These methods are interpretable and need little training data, but each indicator is tied to a particular fault mechanism and machine, and they detect or trend a fault rather than assign it to a severity level learned from data.

Hand-crafted features with shallow classifiers. The second line computes statistical, spectral or time–frequency features and feeds them to a classifier or a statistical model. SVMs have been used to diagnose faults in rotating machinery, power transmission systems, wind turbines and production systems [20,21,22,23] and in hydraulic and oil pumps [24,25], and multivariate statistics of spectral vibration features have been used to identify and quantify hydraulic-system faults [26,27]; neural networks [28] and hidden semi-Markov models [29] have also been applied. For pumps specifically, SVMs were used with empirical mode decomposition and particle-swarm optimisation to predict remaining life from the leakage flow caused by valve-plate wear [30], with chaos features to separate slipper loss from valve-plate wear [31], and with a directed-acyclic-graph structure for five fault conditions of a hydraulic system [32]; more recently, an improved sparse SVM integrated feature selection into the detection of piston wear from forty vibration features [33]. PCA has been used to compress such feature sets and to detect pump faults [2,34]. On the test bench used in the present study, Maradey Lázaro and Borrás Pinilla classified valve-plate wear with wavelet features and artificial neural networks [35,36]. Konieczny and co-workers classified the wear state of a positive-displacement pump with a *k*-nearest-neighbour classifier, using wear produced naturally by many hours of operation with oil of a reduced cleanliness class and checking the classifier on measurements not used for training [37]. This line works with small data sets, and its features can be inspected, but the features and their selection are tuned to one rig, and the reported accuracies are often obtained on test sets drawn from the same acquisition as the training data.

Deep learning. The third line learns the features directly from the signals. Convolutional networks applied to time–frequency images of the vibration, pressure and sound of a hydraulic piston pump identified several fault types [38]; tuning their hyperparameters by Bayesian optimisation [39,40] gave more than 98% accuracy on five typical conditions of an axial piston pump [40]. Deep networks have also been applied to the wear state of a positive-displacement pump [41], and a physics-informed network predicts the pump flow ripple as a health indicator [42]. These models remove the manual design of features and reach high accuracy, but they need large labelled data sets, are harder to interpret, and are usually trained and tested on data from the same rig, operating conditions and acquisition sessions [43].

Transfer learning and multi-source frameworks. The fourth line addresses the dependence on the operating conditions and on the amount of labelled data. Domain-adversarial transfer learning adapts a pump-fault model to new operating conditions [44,45]. Outside hydraulic pumps, multi-source and cross-sensor frameworks have been designed with the same aims: a three-level fusion strategy for the diagnosis of ship motors [46], cross-sensor self-contrastive learning for the fault detection of locomotive motors when labels are scarce [47], and a dual-correlation graph knowledge-distillation network for data-driven bearing prognostics [48]. These frameworks are the most general, but also the most demanding in data and complexity.

Taken together, these studies show that accuracy has increased as features and models have become richer, but three questions have received much less attention. The first is whether a model can grade the severity of a single, well-characterised fault, rather than tell fault types apart. The second is which sensors are actually needed for that fault, which matters because every added sensor raises the cost of a monitoring system. The third is how much of a reported accuracy is retained when the test data differ from the training data. A test on measurements taken after training is reported by Konieczny et al. [37], and generalisation across operating conditions is the aim of the transfer-learning studies [44,45]; a test on a wear state absent from training is, to our knowledge, not reported for the classification of pump wear.

A related, more basic problem concerns how accuracy is estimated. When the training and test sets are drawn from the same recordings—or from overlapping segments of them—the reported accuracy can be optimistic, because the classifier can exploit the fingerprint of each recording rather than the fault itself; this form of data leakage has been documented in bearing-fault benchmarks [49] and across machine-learning-based science [50].

This paper addresses the three questions for valve-plate wear of an axial piston pump. The methods used here—statistical features, principal component analysis (PCA) and a multiclass SVM—are standard, and the focus of the work is on how their results are validated. Using the experimental dataset of Refs. [51,52] (a companion study applied PCA and silhouette analysis to the same fault [53]), we evaluate the same features and classifiers under protocols of increasing difficulty: (i) a record-level, repeated cross-validation within the acquisition campaign, with nested hyperparameter selection, confidence intervals and per-class metrics; (ii) a leave-one-condition-out (LOCO) protocol in which a whole wear state is absent from training, for both a binary decision and a continuous estimate of volumetric efficiency; (iii) a transfer between the two recorded operating points, with and without operating-point normalisation; and (iv) a chronological split within each wear state. The contributions are: (a) a quantitative separation between what these data support as within-campaign separability and what generalises to an unseen wear state; (b) a physical explanation of that separation in terms of the pump’s precharge circuit; and (c) a comparison of pressure, flow and vibration at four pickup positions that identifies a minimal sensor set for this fault. The analysis is limited to a single acquisition campaign, induced wear and two operating points, and the conclusions are stated within that scope. The rest of the paper describes the theoretical background (Section 2), the test bench and the data (Section 3), the results (Section 4), their discussion (Section 5) and the conclusions (Section 6).

## 2. Theoretical Background

### 2.1. Principal Component Analysis

PCA re-expresses a set of correlated features as an ordered sequence of uncorrelated linear combinations—the principal components—each capturing as much of the remaining variance as possible [54]. Working on the standardised feature matrix (every column at zero mean and unit variance, using the training statistics), the components are the eigenvectors of the feature covariance matrix ordered by decreasing eigenvalue $\lambda_{i}$. Retaining the first *a* components, chosen so that

(1) $$\frac{\sum_{i=1}^{a}\lambda_{i}}{\sum_{i=1}^{m}\lambda_{i}}\geq \tau$$

with *m* the number of features and τ a target fraction of retained variance, projects each observation onto an *a*-dimensional subspace. Two uses are made of this projection here: a two-component version for visualising how the wear conditions are arranged in feature space, and a reduction of the feature vector before the classifier, evaluated for τ between 0.5 and 0.99. Since the components are built with no reference to the class labels, PCA may concentrate the discriminative signal or, when that signal lies along low-variance directions, discard it [34,55]. Kernel PCA (KPCA) extends the method to nonlinear structure: the eigen-decomposition is applied to a centred kernel matrix $K(x_{i},x_{j})$ instead of the covariance matrix, so that the components are principal directions in the feature space induced by the kernel [55]; it is also unsupervised.

### 2.2. Support Vector Machines

An SVM [56] is a supervised classifier that, for a training set $\left\{\left(x_{i},y_{i}\right)\right\}$ with $y_{i}\in \left\{-1,+1\right\}$, builds the hyperplane (w·x)+b=0 that maximises the margin between the classes (Figure 1). The margin is 1/∥w∥, so the optimal hyperplane solves

(2) $$\min\frac{1}{2}{∥w∥}^{2}\;s.t.\;y_{i}\left[(w\cdot x_{i})+b\right]\geq 1\;\;\forall i.$$

Introducing Lagrange multipliers $\alpha_{i}\geq 0$ and a penalty *C* for margin violations leads to the dual problem, whose solution gives the decision function

(3) $$f\left(x\right)=\mathrm{sign}\;\left(\sum_{i}\alpha_{i}y_{i}\;\left(x_{i}\cdot x\right)+b\right),$$

in which only the samples with $\alpha_{i}>0$—the support vectors—take part. For non-linearly-separable data, the dot product is replaced by a kernel $K\left(x_{i},x_{j}\right)=\phi {\left(x_{i}\right)}^{⊤}\phi \left(x_{j}\right)$ that performs the mapping and the inner product in one step (Figure 2),

(4) $$f\left(x\right)=\mathrm{sign}\;\left(\sum_{i}\alpha_{i}y_{i}\;K\left(x_{i},x\right)+b\right).$$

The kernels compared here are the linear $K=x_{i}^{⊤}x_{j}$ and the radial basis function (RBF) $K=\exp(-\gamma {∥x_{i}-x_{j}∥}^{2})$, with γ a width parameter.

A multiclass SVM combines several binary classifiers. In the one-against-all strategy, a *k*-class problem is split into *k* binary problems (each class against the rest) and a new sample is assigned to the class whose classifier gives the largest margin (Figure 3a). In the one-against-one strategy, k(k−1)/2 classifiers are trained on pairs of classes and the class is decided by voting; the directed-acyclic-graph variant organises these classifiers in a decision graph to avoid unclassifiable regions [57,58] (Figure 3b). The implementation used here is the error-correcting-output-codes multiclass wrapper of MATLAB R2025a with one-against-one coding.

## 3. Materials and Methods

### 3.1. Test Bench and Instrumentation

The experiments were carried out on a hydraulic test bench built by the Dicbot research group at the Universidad Industrial de Santander for pump-fault characterisation studies [51]. The bench (Figure 4) drives an Eaton series 54 variable-displacement axial piston pump with an electric motor at 1200 rpm. A loading manifold with two crossed relief valves imposes the hydraulic load; the relief setting was fixed at a nominal 700 psi for the loaded tests, so that between tests, the only intended variable is the condition of the valve/bearing plate.

The instrumentation is:

- outlet (port A) pressure: ECO-1 transmitter (WIKA, Klingenberg, Germany), 1–3000 psi, 0–10 V output (calibrated on the bench, $R^{2}=0.9999$);
- precharge pressure: MSP-300 series transducer (Measurement Specialties, Hampton, VA, USA), 1–2500 psi, 0.5–4.5 V output, with an industrial signal-conditioning board ($R^{2}=0.9999$);
- leakage (case-drain) flow: FM-8 turbine flowmeter (Flowmetrics, Chatsworth, CA, USA), $\frac{1}{2}$”, 1–10 gpm, pulse output;
- housing vibration: 352C33 accelerometer (PCB Piezotronics, Depew, NY, USA) (100 mV/g, 0.3–15,000 Hz, resonance >50 kHz), moved between four measurement points (Figure 5): (1) piston barrel, (2)–(3) over the bearing/valve plates, (4) precharge pump.

Signals were acquired with an USB-6215 module (National Instruments, Austin, TX, USA) (16-bit, up to 400 kS/s, ±10 V) for pressure and flow and an USB-9234 dynamic-signal module (National Instruments) (24-bit, ±5 V, anti-aliasing filters) in an NI USB-9162 carrier for vibration, and recorded with a LabVIEW 2013 application. The sampling rate was 100 kS/s for pressure and flow and 50 kS/s for vibration.

### 3.2. Wear Conditions

The loss-of-volumetric-efficiency fault was induced by machining the steel valve plate and the bronze bearing plate, using deep lapping with abrasive paper (grits 120 to 600) and turning on a lathe, until a target efficiency was reached [51] (Figure 6). Because the delivered flow of this pump is not measurable on the bench, the volumetric efficiency of each condition was not measured directly: the original study estimated it indirectly from the increase of the case-drain (leakage) flow and reports it as an approximate value, each condition being targeted at an efficiency band (C1 95–90%, C2 90–85%, C3 85–80%, C4 80–75%, C5 75–65%) [51]; no uncertainty was quantified for these estimates. The average roughness $R_{a}$ of both plates was also measured (SJ-210 profilometer (Mitutoyo, Kawasaki, Japan), eight traces per plate) to follow the machining, but it does not increase monotonically with wear—the valve plate of C4, for instance, has the same $R_{a}$ (0.213 µm) as that of C1, and C4 and C5 share the same bearing-plate $R_{a}$ (2.031 µm) [51]—which indicates that the conditions were produced by different combinations of machined and reused plates rather than by the progressive wear of a single pair. For this reason, roughness cannot serve as an independent check of the efficiency labels. Five conditions were retained for the present analysis, from a nearly new plate to severe wear; a sixth condition prepared with scrapped plates was discarded by the original study because its pressures were not stationary during acquisition, and it is not used here.

Table 1 lists, for each condition, the estimated volumetric efficiency and the campaign-average outlet pressure $P_{A}$, precharge pressure $P_{p}$ and leakage flow $Q_{\ell }$ at the 700 psi operating point. The three quantities vary monotonically with efficiency; note that $P_{A}$ and $P_{p}$ barely change across the first three conditions and then drop sharply at condition 4, a behaviour that is central to the results.

### 3.3. Data Acquisition and Windowing

For each condition, signals were recorded at two operating points: unloaded (neutral) and loaded (≈700 psi), following the protocol of the original study: the bench was run for 10–15 min before each acquisition until the oil reached the working range of 110±10 °F (43±6 °C), monitored with the tank indicator, and about 15 records were taken per variable [51]. All the data were acquired in a single measurement campaign (22–25 February 2016, according to the time stamps embedded in the original file names), and each wear condition corresponds to one mounting of the pump.

The dataset used here contains 15–18 pressure/flow records and 15–19 vibration records per condition, operating point and pickup.

Each record was split into non-overlapping windows: 16,384 samples (≈0.16 s) for pressure/flow and 8192 samples (≈0.16 s) for vibration, with at most 12 windows per record. One window is one observation. Records with non-finite samples were discarded.

### 3.4. Feature Extraction

From every window of every signal, 21 features were computed—the same family of statistical descriptors used in the source MSc study [52]:

- time domain (11): mean level, standard deviation, RMS, peak, peak-to-peak, skewness, kurtosis, and the crest, shape, impulse and clearance factors;
- frequency domain (10, from a Welch periodogram): total power, spectral centroid, spectral spread, spectral kurtosis, the fractional power in the 0–2, 2–5, 5–10, 10–18 and 18–25 kHz bands, and the dominant frequency.

The mean level was taken on the raw window before removing the DC component; all other features were computed on the zero-mean window. Pressure observations thus have 3×21=63 features (outlet pressure, precharge pressure, leakage flow) and vibration observations have 21 features per pickup.

### 3.5. Dimensionality Reduction and Classification

Features were standardised with the mean and standard deviation of the training set of each fold. Classification used a multiclass SVM with one-against-one error-correcting output codes (MATLAB R2025a, fitcecoc) and two kernels: linear and radial basis function (RBF). A random forest (200 trees) and a shallow neural network (two fully connected hidden layers of 20 and 10 units, 500 training iterations) were trained for comparison. Because the pressure and the vibration signals were acquired as separate records, an observation-level fusion of the two modalities was not possible; the pressure features (three channels) and the vibration features (per pickup, and the four pickups pooled) were therefore evaluated as separate feature groups.

Hyperparameters. The SVM hyperparameters were selected by nested cross-validation [59]: inside each outer training set, a three-fold record-level inner cross-validation chose the box constraint $C\in \{10^{-2},10^{-1},\ldots ,10^{3}\}$ for the linear kernel, and $C\in \{10^{-1},\ldots ,10^{3}\}$ together with the kernel scale $s\in \left\{0.25,0.5,1,2,4\right\}\times \sqrt{m}$ (*m* features; $\gamma =1/s^{2}$) for the RBF kernel; the outer test fold was never used for selection. The random forest and the neural network used the fixed settings above. For the LOCO, cross-operating-point, chronological and dimensionality-reduction analyses, a linear SVM with C=1 was used throughout, so that differences between protocols are not confounded with tuning. All random partitions use fixed seeds.

Dimensionality reduction. Principal component analysis was used to visualise the arrangement of the wear conditions in feature space (Section 4). Its effect as a reduction step before the classifier was quantified by retaining the components that explain between 50% and 99% of the variance, and compared with a supervised projection, Fisher’s linear discriminant analysis (LDA) onto four dimensions. In addition, PCA and KPCA (Gaussian kernel, width set to the median squared distance between training observations) were compared for a fixed number of retained components (2–20), each followed by a linear and an RBF SVM. All projections were fitted on the training fold only (Section 5).

Feature selection. No feature selection was applied in the main analyses: every classifier received the full set of 21 features per signal. To assess whether a smaller set would suffice, two filter rankings were computed inside each training fold—the one-way ANOVA *F*-score and minimum-redundancy maximum-relevance (mRMR)—and the linear SVM was retrained on the *k* best-ranked features, for increasing *k*.

### 3.6. Validation Protocols

Four protocols were used, of increasing difficulty:

1. Record-level repeated cross-validation. The records of each condition are assigned to five folds (stratified by condition), so that all the windows of a record fall in the same fold; the procedure is repeated five times with different partitions (25 train/test splits). Accuracy is reported as the mean over the 25 splits with a 95% confidence interval computed with the variance correction of Nadeau and Bengio for overlapping training sets [60]; per-class precision, recall and $F_{1}$ are computed from the pooled confusion matrix, and classifiers are compared with the corresponding corrected resampled *t*-test [60,61]. This measures within-campaign separability of the five wear levels.
2. Leave-one-condition-out (LOCO). The model is trained on four wear conditions and tested on the fifth, which it has never seen. Because a five-class label cannot be predicted for an unseen class, two LOCO targets are used: the binary decision “significant degradation” (volumetric efficiency below 80%, i.e., conditions C4–C5), and the volumetric efficiency itself, estimated as a continuous variable by support-vector and ridge regression. The sensitivity of the binary result to the position of the threshold is examined by moving it to the other band limits. This measures whether the model has learned a transferable property of the fault.
3. Cross-operating-point. The model is trained on the loaded data and tested on the unloaded data, and vice versa, first with the training-set standardisation and then with the features of each operating point standardised by the statistics of the healthy condition C1 recorded at that operating point; in the second case, the test is made on C2–C5, the C1 records of the test operating point being used only as reference. This measures sensitivity to the pump operating point.
4. Chronological split. Within each condition, the records are ordered by acquisition time; the first 70% are used for training and the last 30% for test. This is the closest available proxy for a later measurement of the same state, although all records of a condition were taken within minutes of each other.

## 4. Results

### 4.1. Physical Signature of the Fault

The loss of volumetric efficiency leaves an unambiguous signature in the slow variables (Figure 7). As the efficiency falls from 94.9% to 69.5%, the outlet pressure that the pump can sustain against the fixed relief setting drops from 717 to 484 psi, the precharge pressure from 252 to 101 psi, and the case-drain flow rises from 3.9 to 4.5 gpm. All three trends are monotonic. The pressures change very little among C1, C2 and C3 (a spread of 10 psi in $P_{A}$) and then fall abruptly at C4: the precharge pressure, whose nominal set point is 250 psi, is still near set point for C1–C3 (252–240 psi) and collapses for C4–C5 (131–101 psi). This threshold behaviour—the pump either holds precharge or it does not—organises every result that follows.

### 4.2. Effect on the Vibration Spectrum

The vibration measured over the bearing plate (pickup 2) at the loaded operating point concentrates its energy in a resonance band around 12–13 kHz (Figure 8), in agreement with the original characterisation of this bench [51]. The two most worn conditions (C4, C5) have the highest resonance peaks and sit clearly above the three mild ones from about 13 to 25 kHz, whereas the spectra of C1–C3 are close to one another; this is the same mild/severe grouping that the hydraulic signature shows.

### 4.3. Feature Space and Discriminant Features

Projected onto their first two principal components, the vibration features of the five conditions form two groups separated by a wide gap—the three mild-wear conditions on one side and the two severe ones on the other—rather than five evenly spaced clusters (Figure 9). Within each group, the conditions overlap: C4 and C5 are almost superimposed, and C2 and C3 share most of their range. The first component alone carries 61% of the variance and essentially encodes the mild/severe split.

The most discriminant single features (one–way ANOVA *F*–score over the five conditions, Figure 10) are the overall vibration level (standard deviation and RMS), the spectral centroid, the total power and the spectral spread, followed by the fractional band powers. This is consistent with Figure 8: what changes with wear is how much vibration energy there is and where in frequency it sits.

### 4.4. Within-Campaign Severity Classification

Under record-level repeated cross-validation, with hyperparameters selected in a nested loop, the five wear levels are separated with 94–100% mean accuracy by every classifier and feature group (Table 2). The pressure channels reach 99.6% (95% CI 98.4–100) at 700 psi and 100% in neutral with the linear SVM. Each single accelerometer reaches 98.4–99.8% at 700 psi, and pickup 2 reaches 95.4% in neutral. The differences between the four classifiers are not significant for any single-sensor group (corrected resampled *t*-test, p>0.09), except that for pickup 4 the linear SVM is significantly better than the random forest and the neural network (p=0.014 and p=0.004). Pooling the four accelerometers into a single feature set lowers the linear-SVM accuracy to 94.1% (CI 92.5–95.8), significantly below the RBF SVM, the random forest and the neural network (p≤0.004): the pooled set mixes four differently located sensors whose feature distributions overlap, and only the nonlinear models separate them. The per-class metrics (Table 3) locate the residual errors: at 700 psi they are almost entirely confined to C1/C2 and to C4/C5, the two pairs that are closest in their hydraulic signature (Table 1); in neutral, they mainly concern the three mild states. The same pattern is visible in the pooled confusion matrix of Figure 11.

A chronological split—training on the first 70% of the records of each condition and testing on the last 30%—gives 92.4% (pressure, 700 psi), 100% (pressure, neutral), 100% (pickup 2, 700 psi) and 97.7% (pickup 2, neutral). Within the few minutes over which each condition was recorded, therefore, the separability does not degrade with time; this test cannot, however, reveal differences that would appear between sessions.

### 4.5. Generalisation: Leave-One-Condition-Out

The accuracies above are obtained on records held out from a single acquisition of each wear state, so they measure how separable those five recordings are, not whether a model has learned wear severity. The LOCO analysis tests the latter (Figure 12). For the binary decision “significant degradation” (efficiency below 80%), the pressure channels place every unseen condition on the correct side of the threshold at both operating points (99.7% and 100% mean accuracy; the lowest single value is 98.3%, for C3 at 700 psi). At 700 psi, the vibration of pickups 1, 2 and 3 does the same with 100% for every held-out condition, whereas pickup 4, on the precharge pump, misclassifies most of C3 (39.2%). In neutral, the vibration does not generalise: every pickup fails on at least one held-out condition (for pickup 2, C4 is recognised as degraded in only 31.8% of the windows), and pooling the four pickups at 700 psi also fails on C3 (53.1%).

The position of the threshold matters (Table 4). When the “degraded” group is redefined at the other band limits—C3–C5 (below 87%), C5 alone (below 72%) or C2–C5 (below 92%)—the held-out condition adjacent to the new boundary is misclassified almost completely by the pressure channels and by pickup 2: an unseen C3 is recognised as “below 87%” in 0–1.7% of the windows, and an unseen C4 as “below 72%” in 0%. Only the boundary between C3 and C4 generalises.

### 4.6. Continuous Estimate of the Volumetric Efficiency

Treating the efficiency as a continuous target gives the same picture (Figure 13). Within the campaign, a linear support-vector regressor estimates the efficiency of held-out records with a mean absolute error (MAE) of 0.8 percentage points (pressure, 700 psi; $R^{2}=0.95$) and 1.3 points (pickup 2, 700 psi; $R^{2}=0.96$). Under LOCO, when the regressor must place a condition it has never seen, the MAE rises to 5.4 points for pressure and 4.9 points for pickup 2 (3.6–7.0 points across the four accelerometers), comparable to the 5-point width of the efficiency bands, and the rank correlation between predicted and true efficiency of the five held-out conditions is 0.5–0.8 for all groups except pickup 4 (1.0). The predictions keep the mild group above the severe one, but they do not order C1–C3 (for pickup 2, C3 is predicted at 92.1%, above C2 at 87.9%), and the most worn condition, C5, which requires extrapolation, is overestimated by 6.5 (pickup 2) to 10.1 points (pressure). RBF regression and ridge regression did not improve on the linear SVR (MAE 5.2–10.0 points).

### 4.7. Sensitivity to the Operating Point

A model is strongly tied to the operating point at which it was trained (Table 5). A five-class vibration classifier trained on the loaded data falls to 19.9% on the unloaded data (chance level is 20%), and its binary degradation decision to 59.7%; the reverse direction is worse. The pressure-based models transfer better for the binary decision (76.1% and 94.9%) but not for the five-class one. The neutral and 700 psi records of a given condition were taken minutes apart on the same pump mounting, so these drops are caused by the change of operating point, not by any difference between wear states. Normalising the features of each operating point by the statistics of the healthy condition C1 recorded at that point—a reference that could be collected on a real machine—and evaluating on the remaining conditions C2–C5, raised some binary transfers (pressure and pickup 3, neutral → 700 psi: 100%) but lowered most of the others (for instance pickup 4, neutral → 700 psi: from 81.2% to 49.2%), and it made no five-class transfer reliable (at most 67.9%).

## 5. Discussion

### 5.1. A Physical Threshold Explains the Structure of the Results

The two-group structure seen in Figure 7, Figure 8 and Figure 9 and the LOCO result have a common physical cause. The precharge circuit of this pump needs about 250 psi for the control valve to overcome the servo-piston force and tilt the swashplate to full stroke [51]. For conditions C1–C3, the plates still seal well enough to hold precharge near the set point, the swashplate reaches near-full stroke, and the pump sustains ≈710 psi at the outlet; the three states differ only in fine detail. Between C3 and C4, the leakage crosses the point where precharge can no longer be held: precharge falls to 130 psi and below, the swashplate under-strokes, and the outlet pressure and the vibration signature change abruptly. The classifier therefore has a large, stable, physically grounded boundary to learn (mild vs. significant degradation) and only a subtle one within the mild group. This is exactly what the LOCO analysis reports: the boundary between C3 and C4 generalises to an unseen condition, while boundaries placed anywhere else do not (Table 4), and a continuous regressor keeps the two groups apart but cannot order the states within them (Figure 13).

This also bears on the uncertainty of the efficiency labels. The efficiencies of Table 1 are approximate, indirect estimates with no quantified uncertainty [51], so the five numerical values—and any error expressed in efficiency points, such as the MAE of Section 4—should be read as approximate. The binary result, however, does not depend on those values: the two groups it separates are defined by whether the pump holds its precharge pressure, which is measured directly (240–252 psi versus 101–131 psi). An error of several points in the estimated efficiencies would move the nominal 80% threshold but not the physical boundary between C3 and C4; it would change the name of the threshold, not which conditions fall on each side of it. What the uncertainty does limit is the interpretation of the five-level scale as equally spaced steps of efficiency, which the data do not support in any case.

### 5.2. What the Data Support, and What They Do Not

Within the acquisition campaign, the five wear levels are almost perfectly separable from the pressure signals or from any single accelerometer (Table 2). Taken alone, that number is weak evidence for a diagnostic capability. Each wear condition was recorded once, on one mounting of the pump, with one combination of machined or reused plates, within a few minutes; the records of a condition therefore share not only its wear state but also that mounting, the exact seating of the plates, the placement of the accelerometer and the conditions of that session. A classifier can exploit any such stable difference, wear-related or not, and a record-level cross-validation cannot tell them apart—neither can the chronological split, which spans only minutes. The five-class accuracy is thus a measure of within-campaign separability, and it should not be read as the accuracy that would be obtained on a new mounting, a new session or another pump of the same type.

The LOCO analysis provides stronger evidence for part of the result. The transition to significant loss of volumetric efficiency is detected on a wear state absent from training, from the pressure channels at both operating points and from the vibration of three of the four pickups at 700 psi—so these models have learned a property shared by the degraded conditions, not only the fingerprint of individual recordings. Even this result is bounded by the same campaign: the held-out condition was recorded with the same instruments, the same oil and the same bench as the training conditions, and whether the boundary holds on another mounting or another pump remains to be demonstrated. The fine five-level grading is supported only within the campaign; neither the classifier nor the regressor resolves the mild-wear states of an unseen condition.

### 5.3. Multi-Sensor Reading

For this fault, the pressure channels are the most robust source: they carry the full within-campaign signature, and they are the only feature group whose degradation decision generalises to an unseen condition at both operating points. This is expected, since the pressure signature (Figure 7) is a direct hydraulic consequence of the leakage rather than a learned statistical pattern. Among the accelerometers, the three positions on the piston barrel and over the bearing and valve plates (pickups 1–3) perform alike—within the campaign and under LOCO at 700 psi—whereas pickup 4, on the precharge pump, generalises worse. At the unloaded operating point no accelerometer generalises reliably: each of them fails on at least one held-out condition. Stacking the four pickups into one feature set does not help: it lowers the within-campaign accuracy of the linear model and its LOCO accuracy (87.5% on average, 53.1% for C3, against 100% for pickups 1–3 alone), because it mixes sensors whose features respond differently to wear. The other positions remain useful for other faults (bearing defects, cavitation, precharge-pump faults), which is where multi-sensor fusion pays off [27,37]. Within the scope of this dataset, the most economical instrumentation for detecting significant valve-plate wear on this pump is an outlet- and a precharge-pressure transducer, with one accelerometer on the pump body near the plates as a complementary check at loaded conditions; whether this holds on other mountings and pumps must be verified.

### 5.4. Role of PCA, Kernel PCA and Feature Selection

In this study, PCA serves mainly as an analysis tool: projecting the features on the first two components shows that the five conditions form two groups (Figure 9), with the first component alone (61% of the variance) encoding the mild/severe split—the same structure that the LOCO analysis later reveals. As a reduction step before the classifier, its effect depends on how much variance is retained (Figure 14). For pickup 2 at 700 psi, keeping 95% of the variance (six components) lowers the five-class accuracy from 98.9% to 91.7%, and 99% of the variance is needed to recover 98.5%; for the four pooled pickups, the loss is severe (93.0% to 55.8% at 95%); for the pressure channels, it is small (99.6% to 99.2%). The reason is that PCA orders directions by variance without regard to the labels [34]: the directions that separate the mild-wear states carry little variance and are discarded first. A supervised projection that uses the labels, Fisher’s LDA onto four dimensions, keeps the accuracy of the full feature set for pickup 2 (98.4%) and pressure (99.6%). For the coarse decision with pickup 2 at 700 psi, the reduction is harmless: the LOCO detection of significant degradation stays at 100% for every PCA level tested, because that decision lies along the first, high-variance component. This does not hold for every feature group: with the pressure channels reduced to four components, the LOCO accuracy falls from 99.7% to 86.7% (65.2% for the held-out C4). PCA is therefore useful here to visualise and explain the structure of the data, but not to improve the fine grading or the generalisation, for which the standardised features are used directly.

Table 6 compares PCA and kernel PCA for the same number of retained components, each followed by a linear and an RBF SVM. Kernel PCA does not improve on linear PCA: for a given number of components, it reaches similar or lower accuracy in almost every case, and neither projection exceeds the accuracy of the full feature set. The nonlinear structure that matters for this problem is better handled by the classifier than by the projection: with few components, the RBF SVM recovers much of what the linear SVM loses—for the four pooled pickups, four principal components give 49.0% with the linear kernel and 89.9% with the RBF kernel—while with the full feature set, the gap between the two kernels is much smaller (94.1% and 97.4% for the pooled pickups, and no significant difference for any single sensor; Table 2). Under LOCO, kernel PCA with four components also degrades the detection of significant degradation from the pressure channels (72.8% on average, 16.7% for the held-out C3), whereas with pickup 2 at 700 psi it remains high (98.7%).

The main analyses use the full feature set; Table 7 shows how the accuracy changes when only part of it is kept. When the features are ranked inside each training fold by their ANOVA *F*-score or by mRMR, the eight to twelve best-ranked features already give practically the accuracy of the full set for pressure and for a single accelerometer, while the pooled pickups need almost all of them. The features ranked highest by the *F*-score agree with Figure 10: the level of the outlet and precharge pressures and the spectral shape of the leakage flow for the pressure group, and the vibration level and spectral centroid for the accelerometers. Feature selection thus reduces the cost of the model without changing its accuracy; it does not change the conclusions. Notably, the three best-ranked pressure features alone detect significant degradation on every held-out condition (100% under LOCO), consistent with the physical explanation of Section 5.1.

### 5.5. Operating-Point Dependence and How It Could Be Addressed

The strong dependence on the operating point (Table 5) is expected physically: at the unloaded point, the pump delivers flow against almost no resistance, the precharge circuit is not stressed, and the leakage that defines the fault has little effect on the pressures and the vibration—the neutral-point vibration models do not even generalise across wear conditions at that point. A model trained at one point has thus learned a different mapping from the one needed at the other. Three strategies could reduce this dependence in future work. The first is to include the operating point in the training data, by recording a dense sweep of load pressures (and, ideally, speeds) for every wear state, so that the classifier learns wear-related changes conditional on the operating point. The second is to use features normalised by the operating condition—for instance, deviations from a healthy baseline recorded at the same operating point, or physically normalised quantities such as the ratio of precharge to outlet pressure; the simple healthy-reference normalisation tested here helped only in some transfers, which suggests that a baseline must be learned as a function of the operating point rather than stored at two points. The third is transfer learning and domain adaptation, in which a model trained on labelled data at one condition is adapted, with little or no labelled data, to another [44,45], or multimode monitoring schemes that first identify the operating mode [55]. Evaluating these strategies requires more operating points than the two available here.

### 5.6. Induced Versus Natural Wear

The wear states were produced by lapping and turning the plates and, as the roughness data show, by combining machined and reused plates [51]. This allows controlled, well-separated states, but it differs from natural wear in several ways: natural wear is progressive and non-uniform, concentrated where the sealing lands are most loaded; it is accompanied by the wear of other parts (slippers, swashplate, bearings); and it evolves together with the oil condition and the operating history. A model trained on machined states may therefore rely on features—for example, a particular pattern of leakage between the kidney ports or a particular seating of a reused plate—that natural wear does not reproduce, and it has not been tested on naturally worn components. The coarse boundary found here is defined by a hydraulic consequence (the loss of precharge pressure) that any sufficiently advanced valve-plate wear should produce, which gives some reason to expect it to transfer better than the fine grading; this expectation, however, remains to be verified. Studies in which the wear develops naturally during long operation, as in Ref. [37], are the necessary next step.

### 5.7. Potential Confounding Factors

Because each wear condition coincides with one mounting and one short recording session, any factor that varied between sessions could contribute to the separability, and several can be assessed from the acquisition records. Oil temperature was held within 110±10 °F after a 10–15 min warm-up [51]; a spread of 20 °F changes the oil viscosity and hence the leakage, so part of the difference between neighbouring mild states could be thermal, and the temperature of each record was not stored. Acquisition order does not follow the wear order: according to the time stamps of the original files, C1 was recorded on 22 February, C2, C5 and C4 (in that order) on 23 February, and C3 on 25 February; a slow drift of the bench over the campaign would therefore not reproduce the ordering of the wear states. Mounting and plate seating changed with every condition, and some plates were reused across conditions; this confound cannot be separated from the wear state with the available data and is the main reason why the within-campaign accuracy cannot be read as cross-session performance. Accelerometer placement was repeated at each of the four points for each condition, so small differences in the mounting of the sensor would affect the vibration features but not the pressure features; the fact that the pressure channels generalise at least as well as the vibration under LOCO suggests that sensor placement is not the main source of separability. Fluid condition (contamination, ageing) was not monitored; since the campaign lasted four days, large changes are unlikely, but they cannot be excluded.

### 5.8. Comparison with the Literature

Table 8 places the results next to those of the two most closely related wear-classification studies for which both the validation protocol and the numerical results are reported in full [35,37]. Direct comparison is limited because the pumps, the faults, the number of classes and—most importantly—the validation protocols differ. The closest reference is the work of Maradey Lázaro and Borrás Pinilla [35], carried out on the same test bench and the same set of wear conditions—including the sixth, 64.9% condition that is excluded here—with wavelet-packet features of the vibration signals and several neural-network classifiers; on a stratified 70/30 split they report test accuracies of 83.3–95.8%, of the same order as the within-campaign accuracies of Table 2. The difference lies in what is additionally reported. Konieczny et al. [37] worked with naturally developed wear and verified their classifier on new measurement data, with a drop from 94.5% (cross-validation) to 90%; the present study cannot offer a comparable test on new data, but it quantifies generalisation to a wear state absent from training, which is not reported by the studies compared in Table 8; generalisation across operating conditions has been addressed with transfer learning by Dong et al. [44]. What the present study adds is therefore not a higher accuracy, but a separation between what generalises and what reflects only within-campaign separability.

### 5.9. Limitations

The main limitations of the study are the following. (i) Single campaign, one mounting per condition. All the data come from one acquisition campaign, and each wear condition corresponds to one mounting of the pump, with its own combination of plates; the models may therefore capture the configuration, sensor response and conditions of each session together with the wear. A between-session validation—disassembly, remounting and re-recording of the same wear state, ideally on another day and another pump—is necessary before any of the within-campaign accuracies can be read as diagnostic performance, and it is the most important direction for future work. (ii) Two operating points. Models do not transfer between the loaded and unloaded points, and two points are not enough to characterise or correct this dependence. (iii) Indirect, approximate labels. The volumetric efficiency was estimated from the leakage flow and has no quantified uncertainty; the class boundaries between neighbouring mild states are correspondingly uncertain, although the C3/C4 boundary is fixed by the directly measured precharge pressure. (iv) Induced wear. Machined and reused plates may not reproduce natural wear, and no naturally worn component was tested. (v) Unrecorded covariates. The oil temperature of each record, the oil condition and the exact sensor mounting were not stored. (vi) Finally, a single fault mode is considered, the processing is offline, and the pressure and vibration signals were recorded separately, which prevented an observation-level fusion of the two modalities.

## 6. Conclusions

Pressure, flow and vibration signals recorded on a test bench in a single acquisition campaign were used to study how far valve-plate wear—and the associated loss of volumetric efficiency—can be detected and graded in an Eaton series 54 axial piston pump with standard statistical features and classifiers. All the conclusions below refer to this dataset: five induced wear states, each recorded on one mounting of the pump, at two operating points.

- The loss of volumetric efficiency has a clear hydraulic signature: as efficiency falls from 94.9% to 69.5%, outlet and precharge pressure drop and leakage flow rises, with a sharp step once the precharge pressure can no longer be held.
- Within the acquisition campaign, the five wear states are separable with 94–100% accuracy (record-level cross-validation) from the pressure channels or from any single accelerometer. Because each state was recorded once, on one mounting, this is a measure of within-campaign separability, not of diagnostic accuracy on new mountings, sessions or pumps.
- Under leave-one-condition-out validation, only the coarse decision generalises: the transition to significant degradation (efficiency below about 80%, where the pump loses its precharge pressure) is detected on an unseen wear state by the pressure channels at both operating points and by the vibration of three of the four pickups at the loaded point. Neither the fine ordering of the mild-wear states nor a continuous estimate of the efficiency (mean absolute error 3.6–7.0 points) generalises to an unseen state.
- Models are tied to the operating point at which they were trained, and a simple healthy-reference normalisation does not remove this dependence.
- PCA is useful to visualise and explain the two-group structure of the data, but reducing the features before the classifier degrades the fine grading unless almost all the variance is retained.

Within this scope, the results indicate that the loss of precharge pressure associated with significant valve-plate wear can be recognised from pressure transducers, or from an accelerometer on the pump body at a loaded operating point, on a wear state not seen in training. Whether this holds on other mountings, other acquisition sessions, other operating points, naturally worn components and other pumps of the same type has not been demonstrated; a multi-session, multi-operating-point experiment on naturally worn components is the necessary next step before any use for condition-based maintenance.

## Figures and Tables

**Figure 1:**
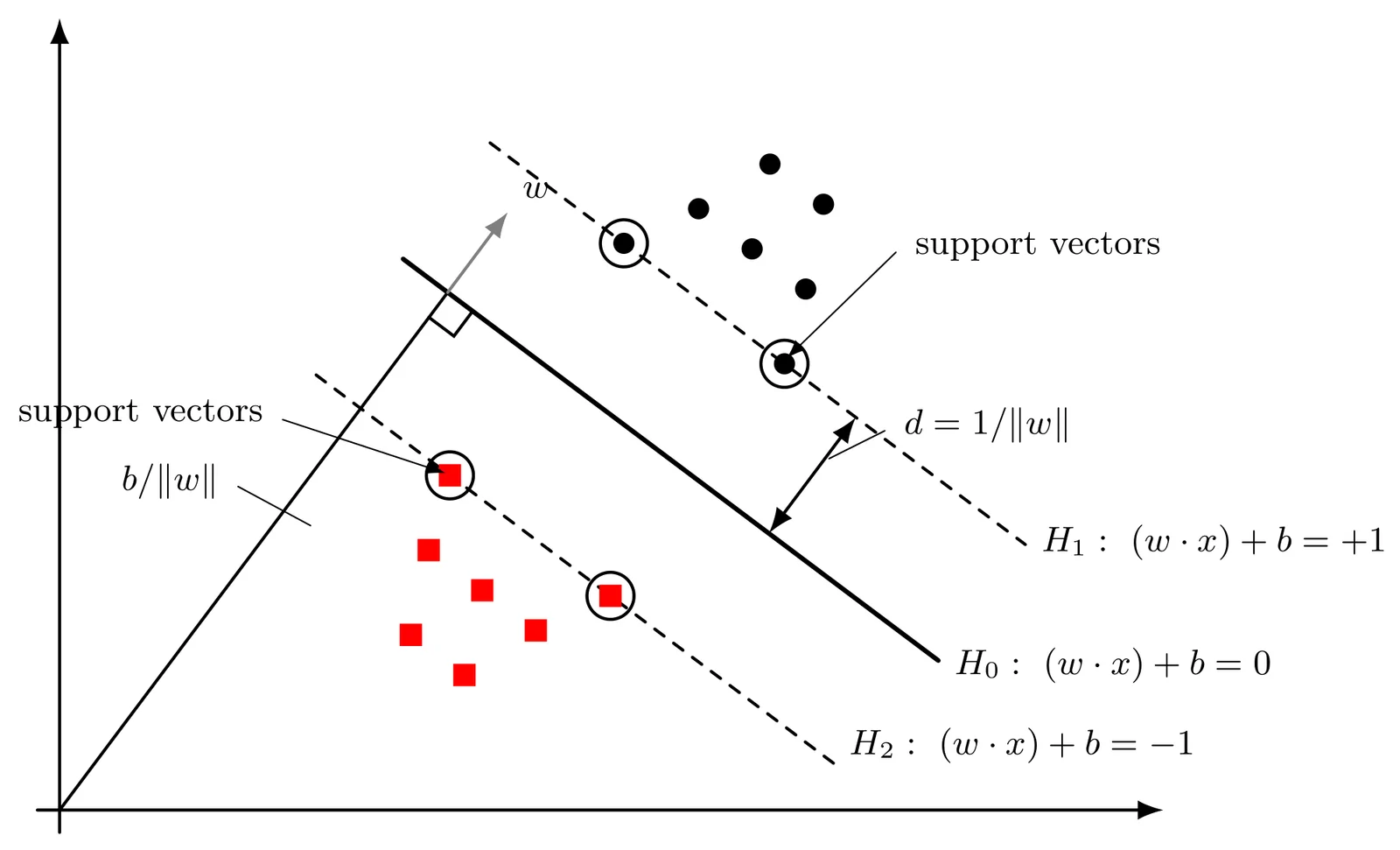
Optimal hyperplane and margin maximisation for a two-class SVM.

**Figure 2:**
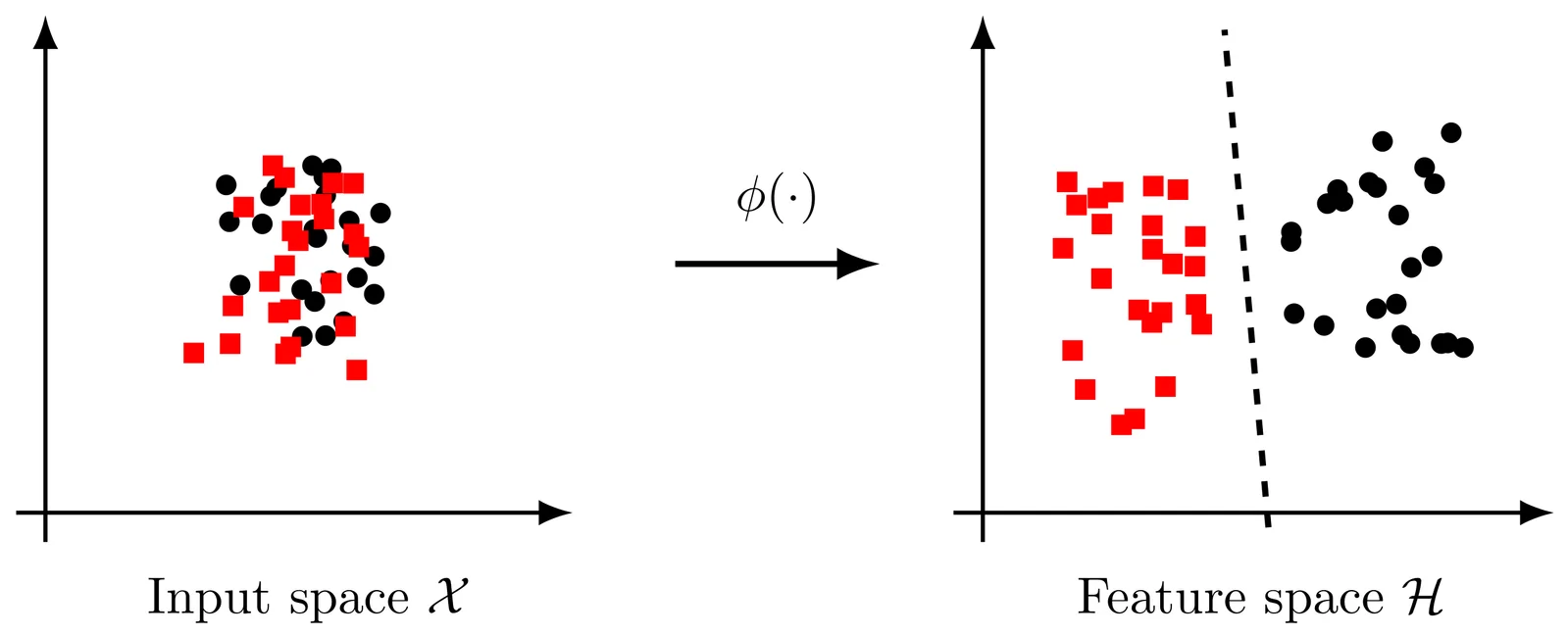
A kernel maps the data into a space where the classes become linearly separable.

**Figure 3:**
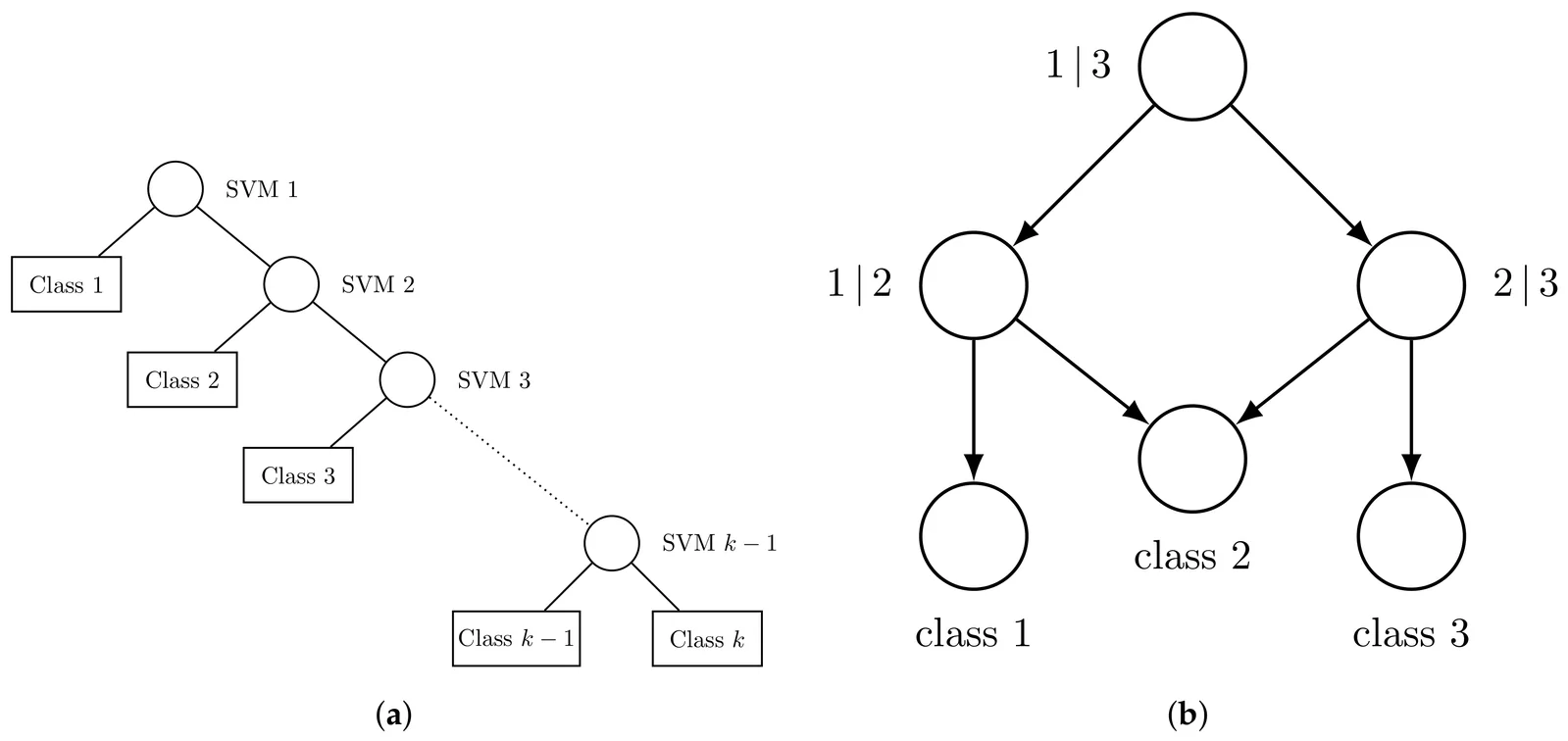
Multiclass SVM structures: (**a**) one-against-all cascade; (**b**) directed-acyclic-graph decision structure for three classes.

**Figure 4:**
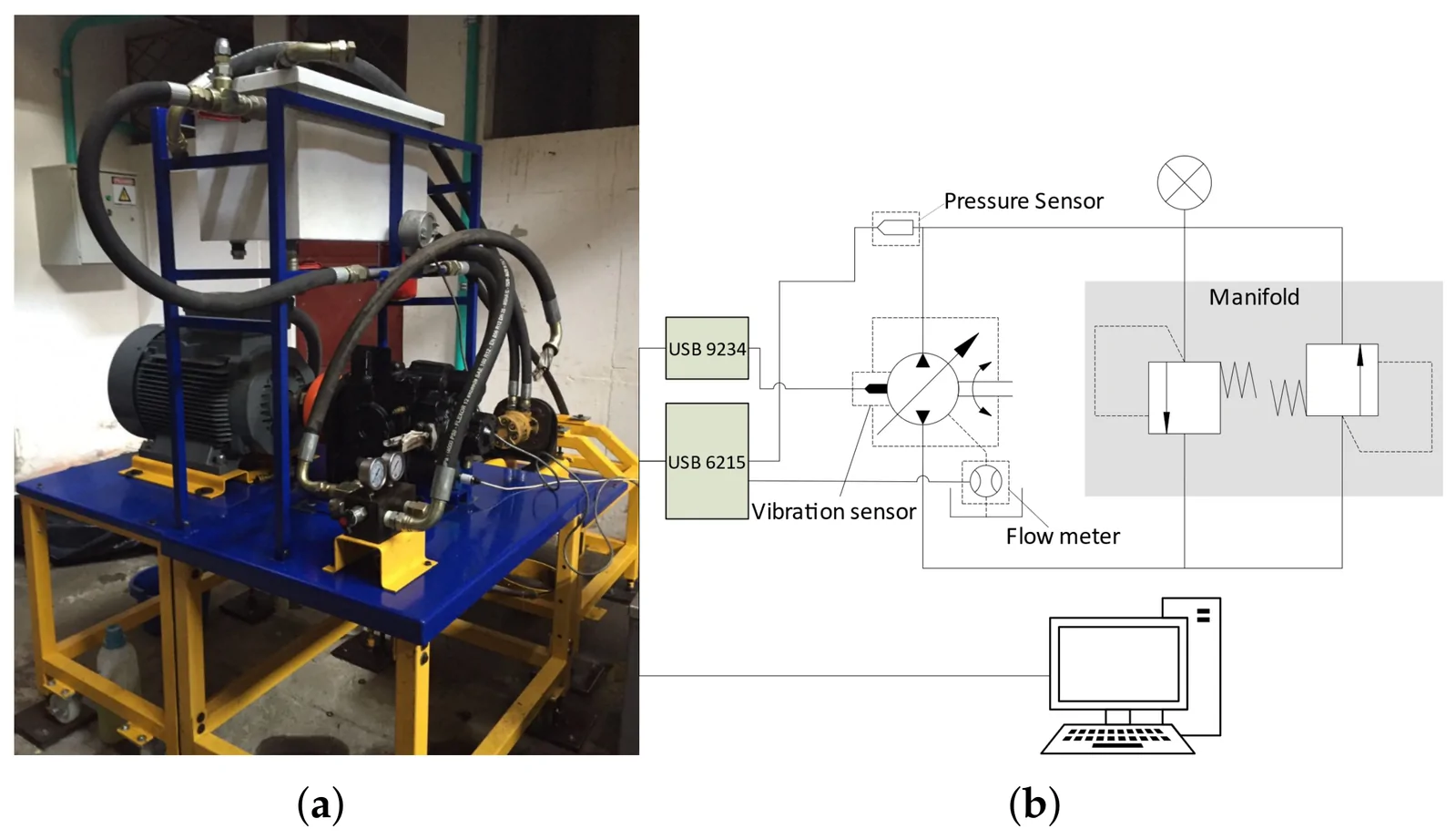
Test bench used for the experiments: (**a**) real bench; (**b**) hydraulic schematic.

**Figure 5:**
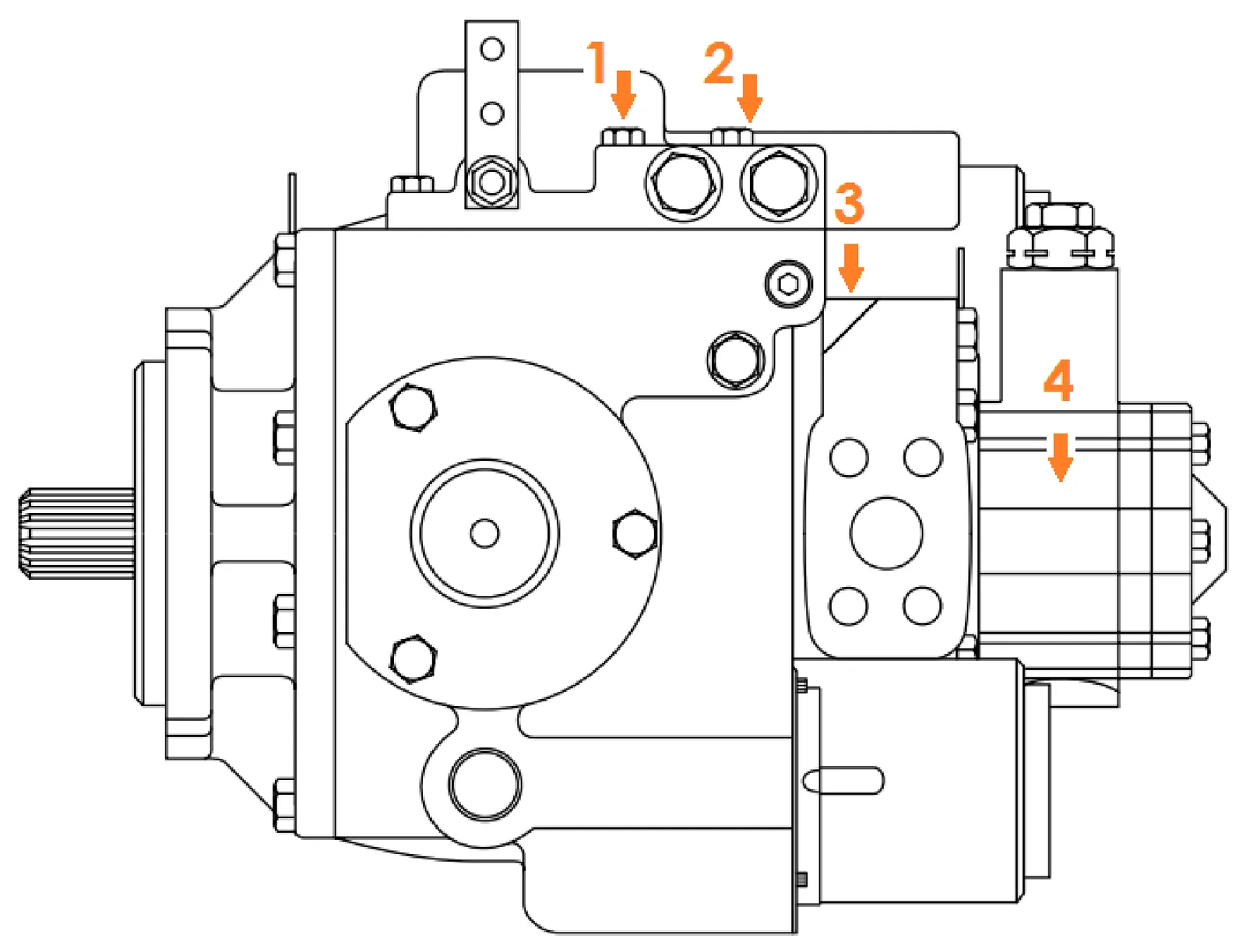
Vibration measurement points on the pump housing: (1) piston barrel, (2)–(3) over the bearing and valve plates, (4) precharge pump.

**Figure 6:**
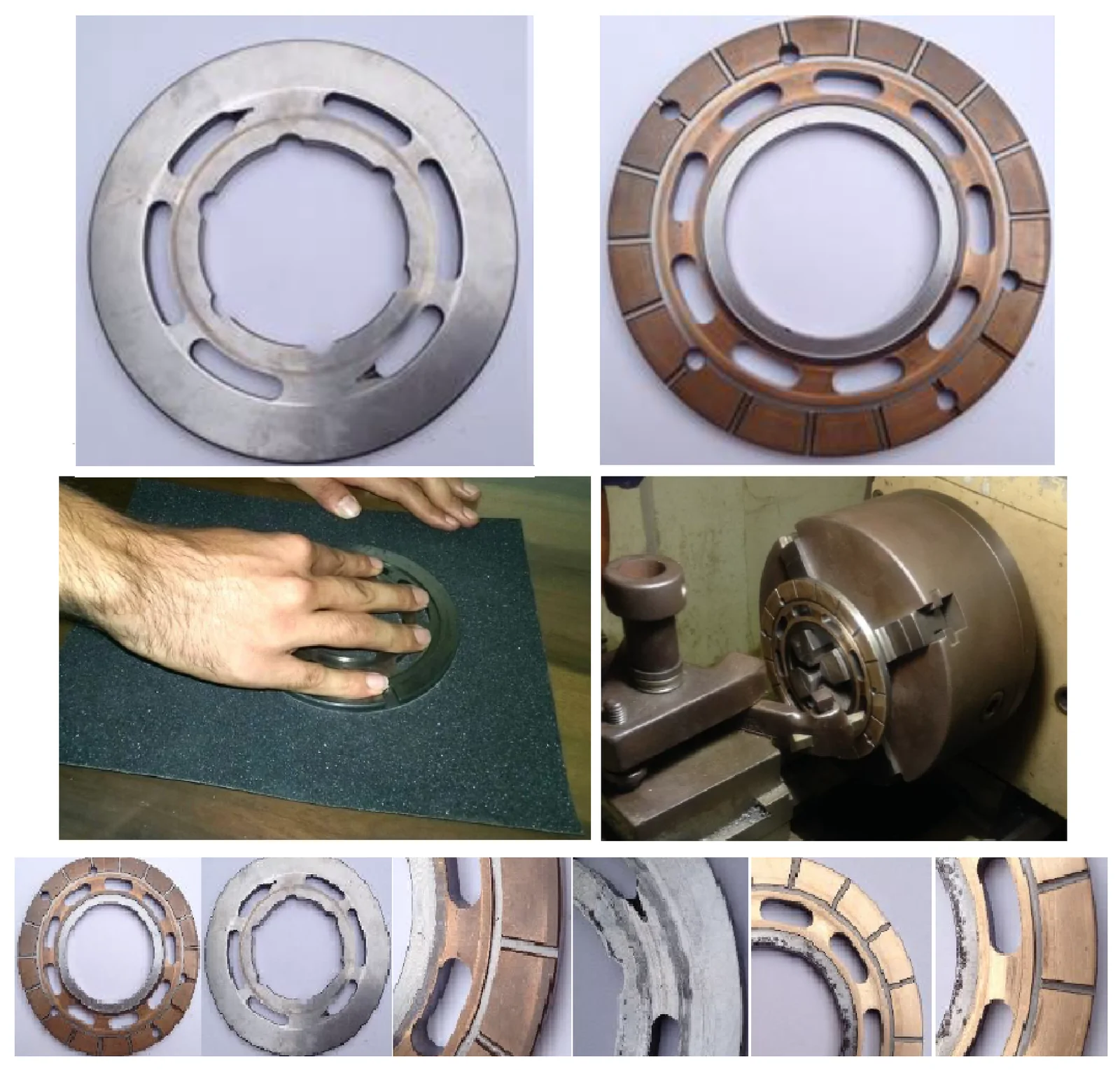
Valve and bearing plates of the pump, and the deep lapping and lathe turning used to machine them to the target efficiencies; the bottom row shows the resulting wear on a bearing plate.

**Figure 7:**
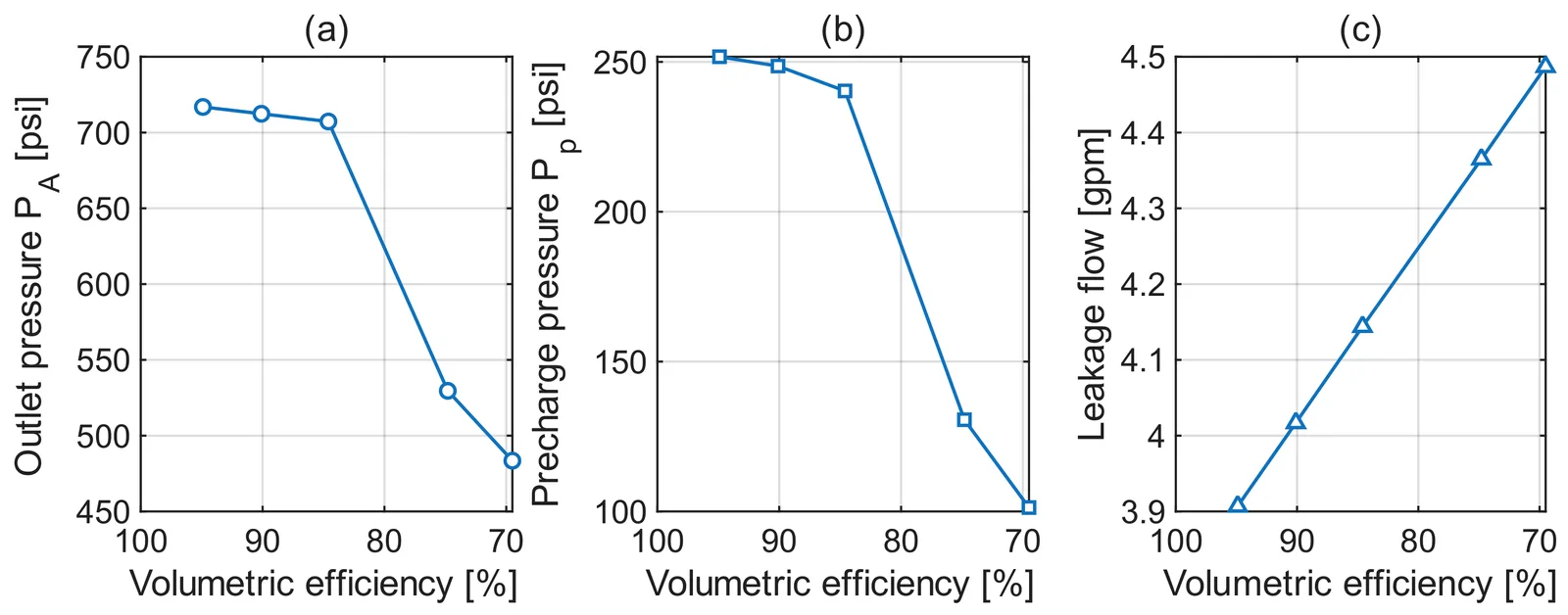
Hydraulic signature of the fault at the 700 psi operating point: (**a**) outlet pressure, (**b**) precharge pressure and (**c**) leakage flow versus volumetric efficiency. The pressures are nearly constant for the three mildest wear states and then drop sharply.

**Figure 8:**
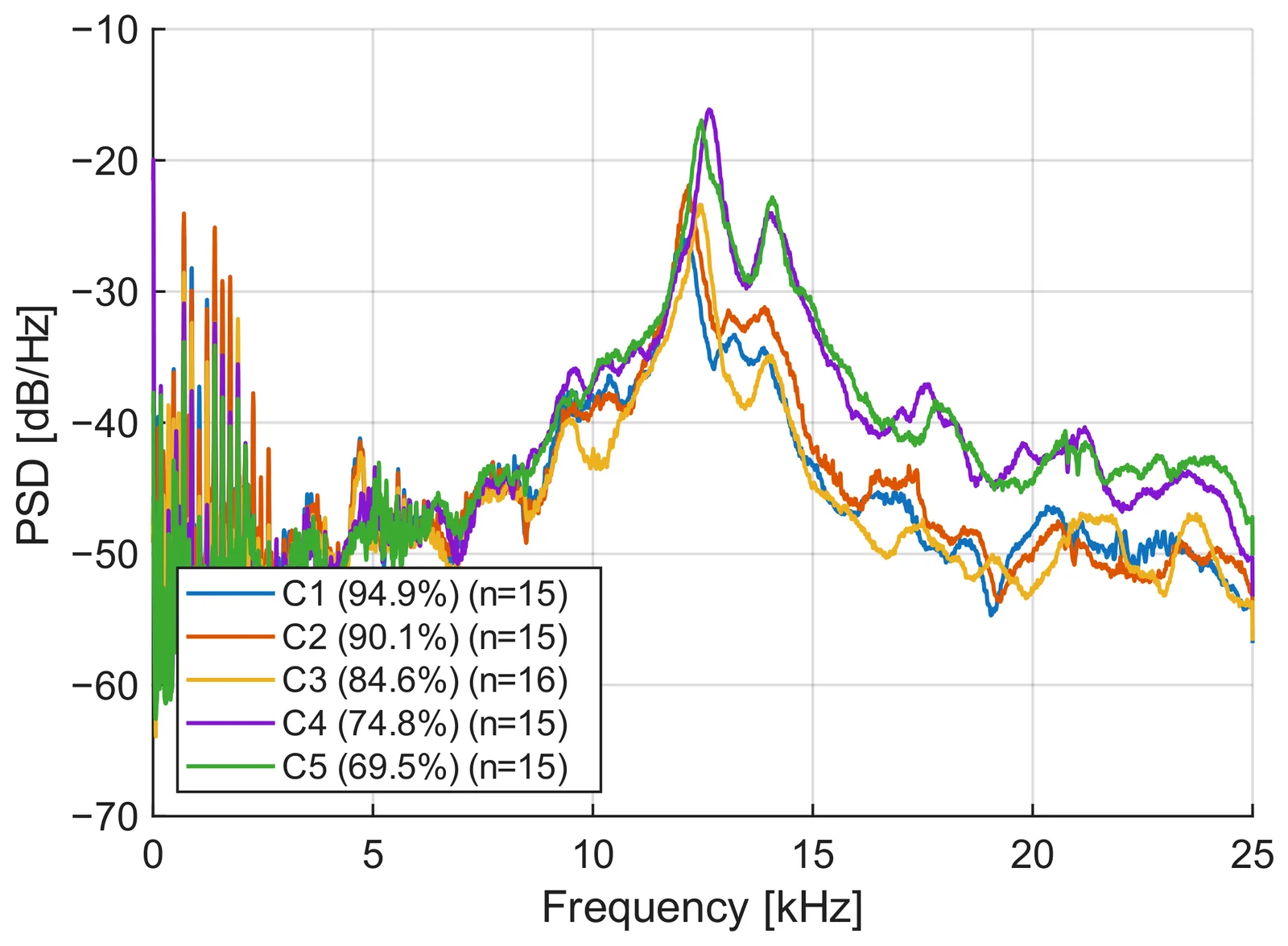
Power spectral density of the bearing–plate vibration (pickup 2, 700 psi) for the five wear conditions, averaged over the *n* unique records of each condition (Welch estimate, 4096–point Hann window, 50% overlap). Energy in the 12–25 kHz band is higher for the two most worn conditions.

**Figure 9:**
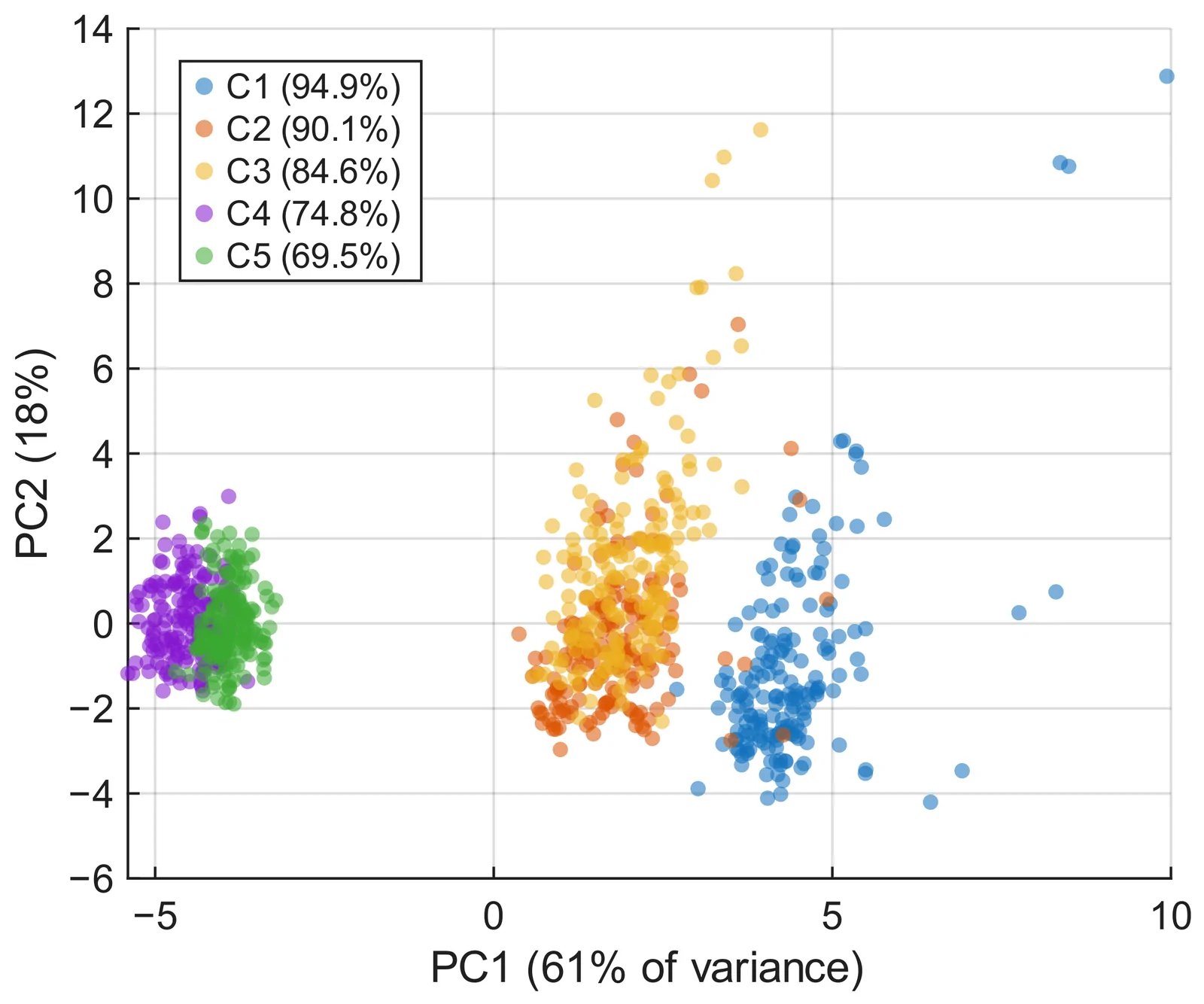
First two principal components of the bearing–plate vibration features (pickup 2, 700 psi). The five wear conditions form two groups separated by a gap—three mild-wear states and two severe ones—rather than five evenly spaced clusters.

**Figure 10:**
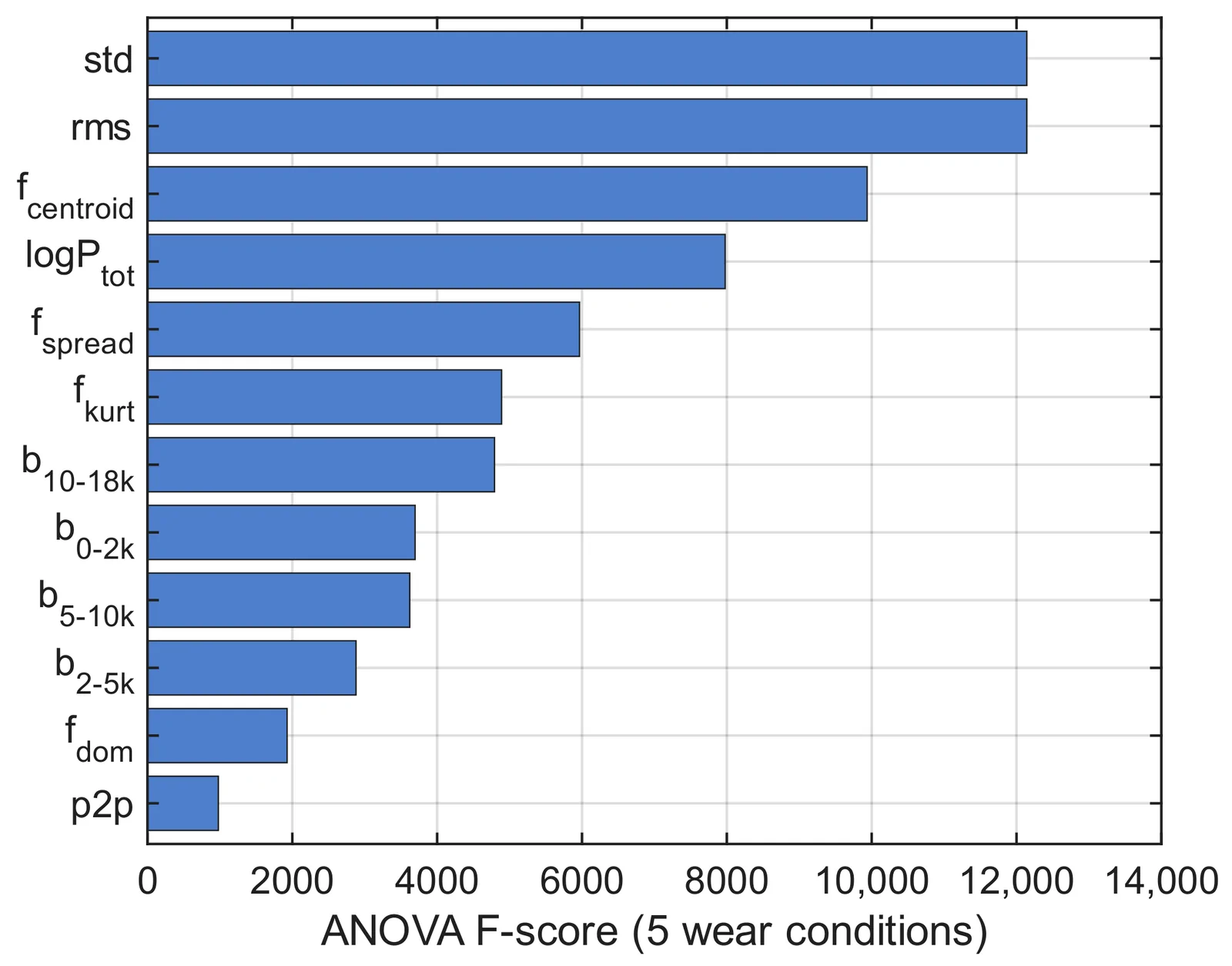
Discriminant power (one-way ANOVA *F*-score over the five wear conditions) of the twelve most informative bearing-plate vibration features.

**Figure 11:**
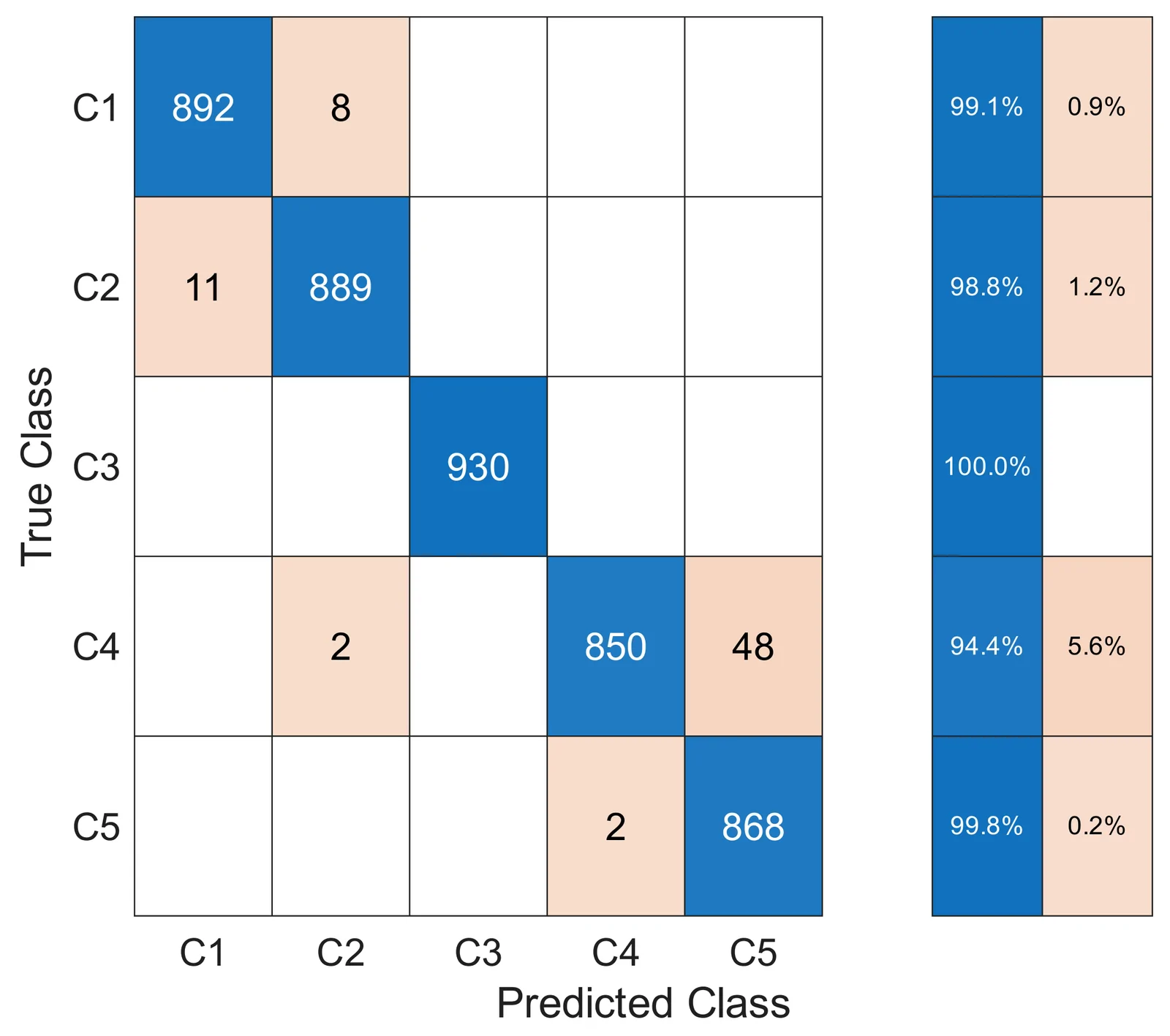
Confusion matrix of the five-class linear SVM on the bearing-plate vibration (pickup 2, 700 psi), pooled over the 25 record-level cross-validation test folds; the right-hand column gives the per-class recall.

**Figure 12:**
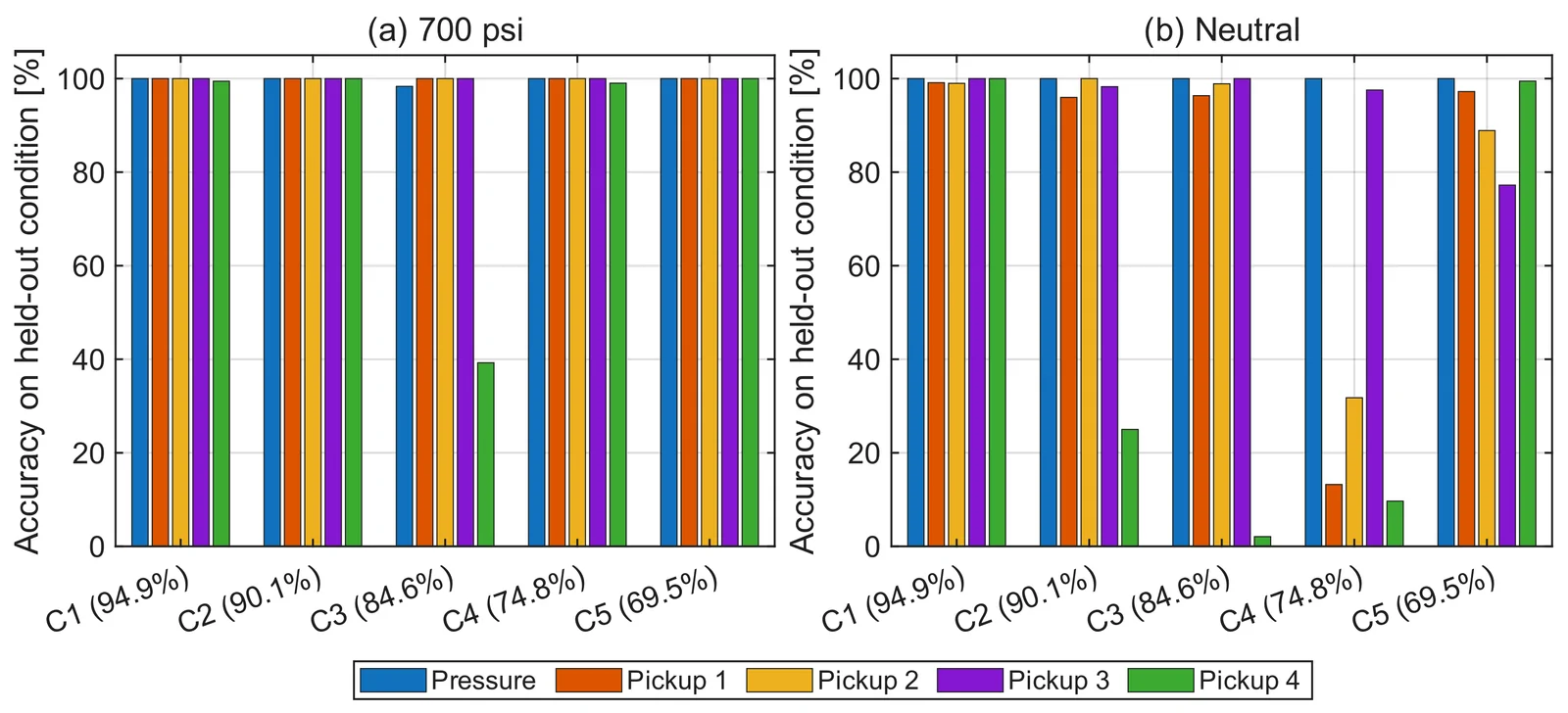
Leave-one-condition-out detection of significant degradation (efficiency < 80%): accuracy on each wear condition when it is excluded from training, for the pressure channels and each accelerometer, at (**a**) 700 psi and (**b**) the neutral operating point (linear SVM, C=1).

**Figure 13:**
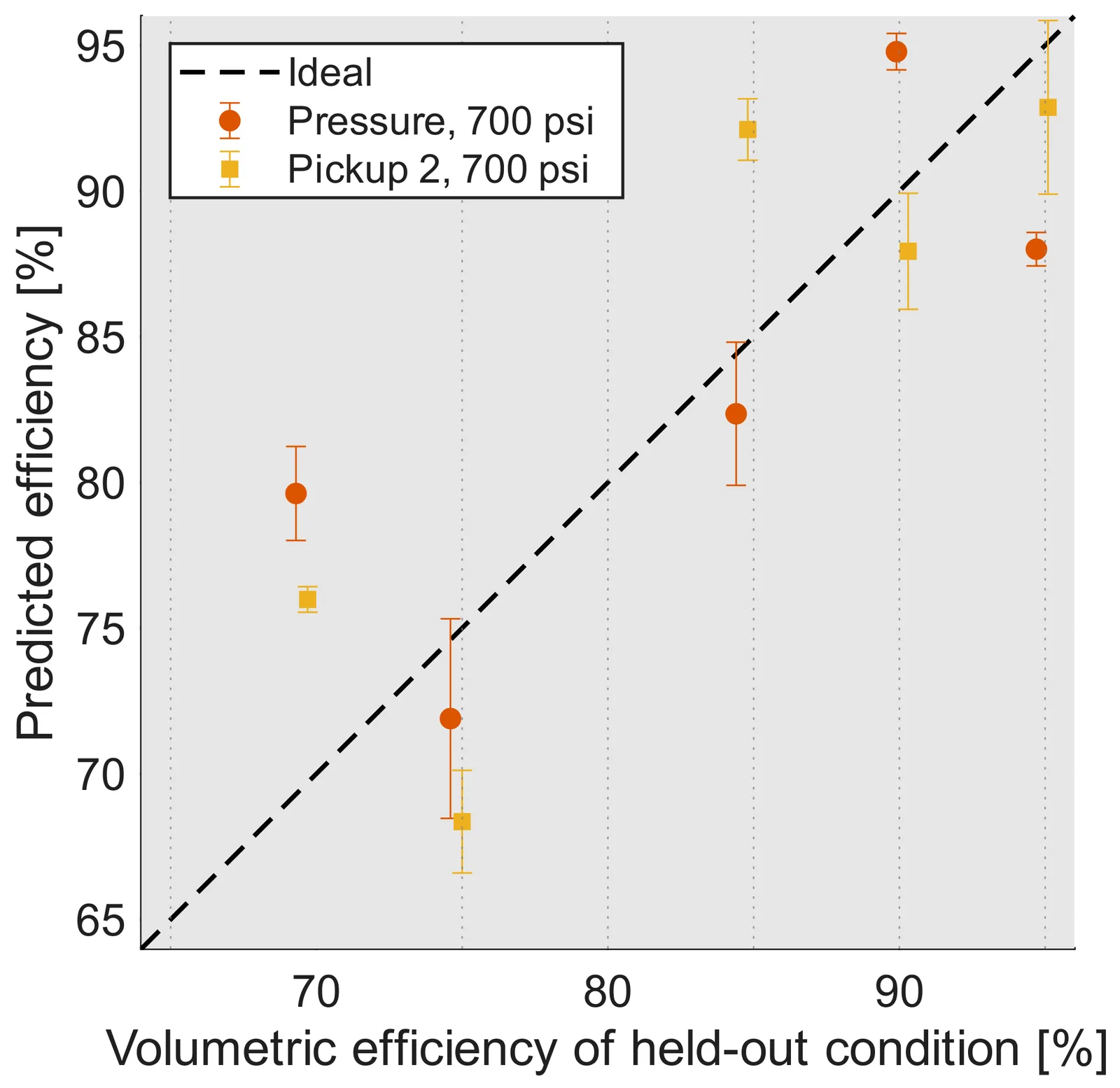
Leave-one-condition-out estimate of the volumetric efficiency (linear SVR): mean ± standard deviation of the prediction for each held-out condition, against its nominal efficiency. The dotted lines mark the efficiency bands of the original study.

**Figure 14:**
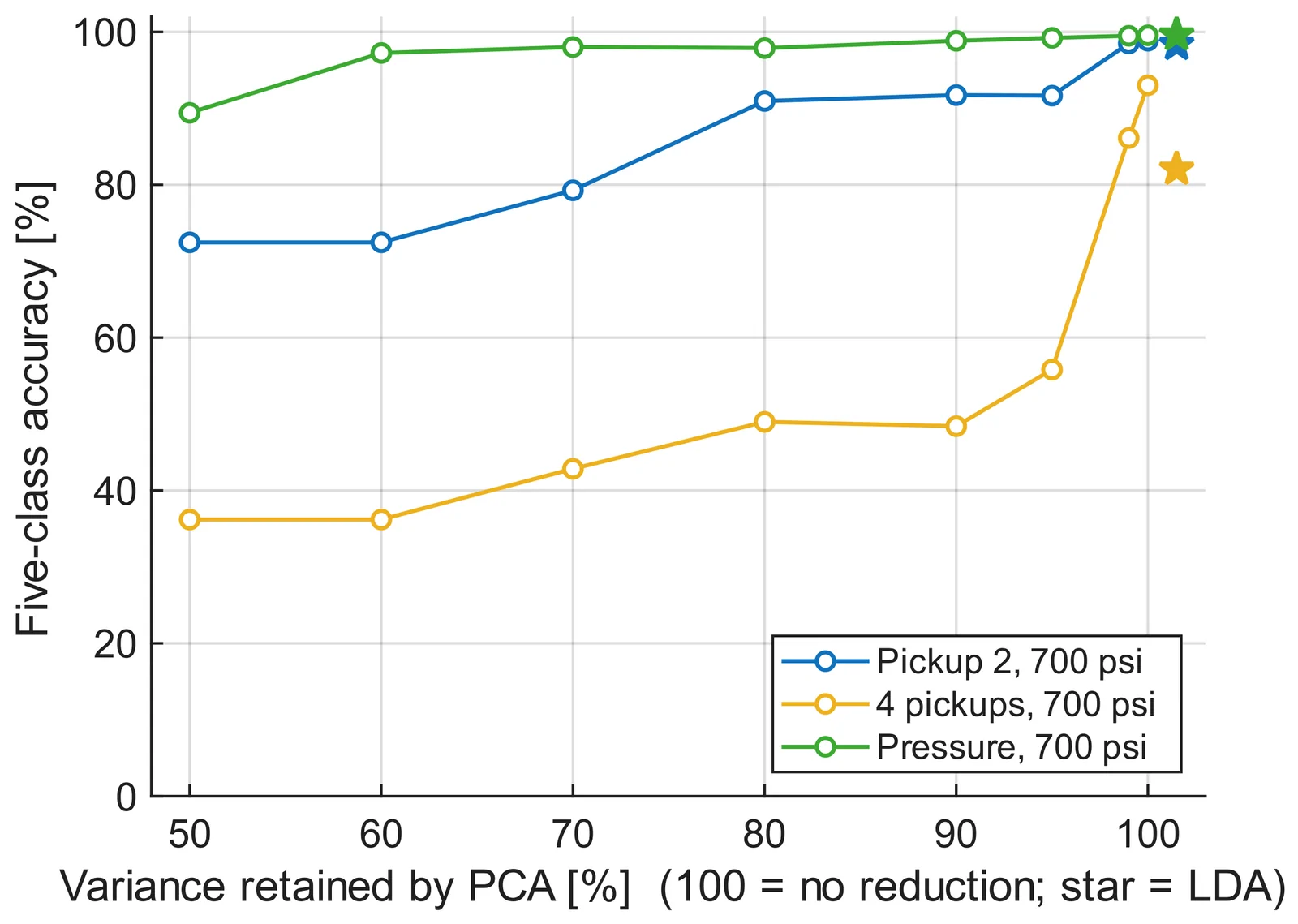
Five-class accuracy of the linear SVM (C=1, record-level cross-validation) after PCA reduction, as a function of the variance retained (100% = no reduction); the stars give the accuracy after a supervised LDA projection onto four dimensions.

**Table 1:** Wear conditions and their measured hydraulic signature at the 700 psi operating point (values after Ref. [51]).

Cond.	Volumetric Efficiency [%]	$P_{A}$ [psi]	$P_{p}$ [psi]	$Q_{\ell }$ [gpm]
C1	94.9	717	252	3.91
C2	90.1	712	249	4.02
C3	84.6	707	240	4.14
C4	74.8	530	131	4.37
C5	69.5	484	101	4.49

**Table 2:** Five-class classification accuracy [%] under record-level cross-validation (5 folds × 5 repetitions; mean, with the 95% confidence interval of the linear SVM in brackets). SVM hyperparameters selected by nested cross-validation.

Op. Point	Feature Group	SVM Linear	95% CI	SVM RBF	Random Forest	Neural Net
700 psi	Pressure (3 channels)	99.6	[98.4, 100]	99.2	100	98.6
Neutral	Pressure (3 channels)	100	[100, 100]	99.5	100	99.8
700 psi	Vibration, pickup 1	98.4	[96.5, 100]	96.9	97.1	96.5
700 psi	Vibration, pickup 2	98.4	[95.9, 100]	98.0	99.7	97.6
700 psi	Vibration, pickup 3	99.8	[99.5, 100]	98.7	99.3	99.7
700 psi	Vibration, pickup 4	98.5	[97.3, 99.7]	97.4	95.8	95.6
700 psi	Vibration, 4 pickups pooled	94.1	[92.5, 95.8]	97.4	98.1	96.9
Neutral	Vibration, pickup 2	95.4	[92.4, 98.4]	94.7	94.5	95.1

**Table 3:** Per-class recall and $F_{1}$ [%] of the linear SVM (pooled over the 25 cross-validation test folds).

Feature Group	C1	C2	C3	C4	C5
Recall					
Pressure, 700 psi	100	100	100	99.1	99.0
Pickup 2, 700 psi	99.1	98.8	100	94.4	99.8
Pickup 2, neutral	92.1	95.7	90.6	98.9	100
$F_{1}$					
Pressure, 700 psi	100	100	100	99.1	99.0
Pickup 2, 700 psi	98.9	98.8	100	97.0	97.2
Pickup 2, neutral	92.0	95.5	90.6	99.4	99.7

**Table 4:** LOCO binary accuracy [%] averaged over the five held-out conditions, for different positions of the degradation threshold; in brackets, the worst held-out condition. Linear SVM, 700 psi.

“Degraded” Group	Pressure	Pickup 2	4 Pickups Pooled
C4–C5 (efficiency < 80%)	99.7 (C3: 98.3)	100 (100)	87.5 (C3: 53.1)
C3–C5 (<87%)	80.0 (C3: 1.7)	78.4 (C3: 0)	75.7 (C3: 46.9)
C5 (<72%)	75.0 (C4: 0)	75.0 (C4: 0)	75.4 (C4: 10.2)
C2–C5 (<92%)	52.1 (C2: 0)	65.9 (C2: 1.1)	59.1 (C2: 28.6)

**Table 5:** Cross-operating-point accuracy [%] (linear SVM, C=1). Left: features standardised with the training data, test on C1–C5. Right: features of each operating point standardised with its own healthy reference C1, test on C2–C5 (the C1 records of the test operating point are used only as reference).

Feature Group	Train → Test	Training Standardisation		Healthy (C1) Reference	
		Binary	Five-Class	Binary	Five-Class
Pressure	700 → neutral	76.1	19.9	74.4	21.8
Pressure	neutral → 700	94.9	42.5	100	13.5
Pickup 2	700 → neutral	59.7	19.9	48.8	1.2
Pickup 2	neutral → 700	30.3	6.8	28.3	15.7
Pickup 3	700 → neutral	75.8	32.7	55.2	24.4
Pickup 3	neutral → 700	92.7	58.4	100	67.9

**Table 6:** Five-class accuracy [%] for a fixed number of components retained by PCA and by kernel PCA (Gaussian kernel), followed by a linear or an RBF SVM (C=1; record-level cross-validation, 5 folds × 5 repetitions). “All” = no reduction.

Feature Group	Components	PCA + Linear	PCA + RBF	KPCA + Linear	KPCA + RBF
Pressure, 700 psi	2	89.4	89.9	90.1	92.3
	4	98.0	97.1	95.7	96.5
	8	98.8	97.9	97.4	96.8
	20	99.5	98.5	97.5	98.0
	All (63)	99.6	98.5	–	–
Pickup 2, 700 psi	2	79.3	75.6	62.4	69.5
	4	91.0	91.3	86.1	86.6
	8	98.5	97.4	95.1	95.0
	20	98.9	97.4	97.4	97.6
	All (21)	98.9	97.4	–	–
Pickup 2, neutral	4	88.2	87.2	83.0	84.0
	8	93.7	91.6	88.8	89.1
	All (21)	95.4	92.6	–	–
4 pickups pooled, 700 psi	4	49.0	89.9	39.5	87.0
	8	69.6	91.3	61.2	90.2
	20	93.0	96.3	78.4	95.4
	All (21)	93.0	96.2	–	–

**Table 7:** Five-class accuracy [%] of the linear SVM (C=1) trained on the *k* best-ranked features, with the ranking computed inside each training fold by the ANOVA *F*-score (F) or by mRMR (M); record-level cross-validation, 5 folds × 5 repetitions.

*k*	Pressure, 700 psi		Pickup 2, 700 psi		Pickup 2, Neutral		4 Pickups, 700 psi	
	F	M	F	M	F	M	F	M
3	95.3	93.6	86.6	92.4	84.8	84.2	52.5	43.6
6	97.4	98.4	93.5	94.8	92.2	93.2	64.0	56.5
8	99.2	99.6	97.5	98.1	94.3	94.2	81.3	69.0
12	100	99.3	98.9	99.0	96.0	94.5	85.3	84.2
All	99.6	99.6	98.9	98.9	95.4	95.4	93.0	93.0

**Table 8:** Comparison with related wear-classification studies of piston pumps. “Validation” states how each reported accuracy was obtained.

Study	Pump/Fault	Signals, Method	Validation	Reported Accuracy
Maradey Lázaro and Borrás Pinilla [35]	Axial piston pump, same bench and wear conditions as this work; induced valve-plate wear, 6 states	Vibration; wavelet-packet features; Adaline, nonlinear and MLP networks	Stratified 70/30 train/test split	83.3–95.8% (test)
Konieczny et al. [37]	Multi-piston pump; natural wear, 3 states	Vibration; *k*-NN	Cross-validation; verification on new measurements	94.5% (CV); 90% (new data)
This work	Axial piston pump; induced valve-plate wear, 5 states	Pressure, vibration; SVM	Record-level CV	94.1–100%
			LOCO, binary (<80%)	99.7–100% (pressure); 100% (pickups 1–3, 700 psi)
			LOCO, continuous efficiency	MAE 3.6–7.0 points
			Cross-operating-point	30–95% (binary)

## Data Availability

The experimental dataset was acquired on the UIS Dicbot hydraulic test bench and is described in Ref. [51]. The processed feature files and the analysis scripts that support the findings of this study may be made available by the corresponding author upon reasonable request, subject to the authors’ assessment of each request.
